# Barriers and facilitators to the implementation of PHAROS, a perioperative pharmaceutical management intervention for older adults – a qualitative interview study from the perspective of healthcare providers

**DOI:** 10.1186/s12877-024-05652-4

**Published:** 2025-01-21

**Authors:** Moritz Sebastian Schönfeld, Julia Rinke, Claudia Langebrake, Levente Kriston, Cynthia Olotu, Rainer Kiefmann, Corinna Bergelt

**Affiliations:** 1 Department of Medical Psychology, University Medical Center Hamburg-Eppendorf, Martinistraße 52, 20246 Hamburg, Germany; 2 Hospital Pharmacy, University Medical Center Hamburg-Eppendorf, Martinistraße 52, 20246 Hamburg, Germany; 3 Department of Stem Cell Transplantation, Hospital Pharmacy, University Medical Center Hamburg-Eppendorf, Martinistraße 52, 20246 Hamburg, Germany; 4 Department of Medical Psychology, University Medicine Greifswald, Walther-Rathenau-Straße 48, 17475 Greifswald, Germany; 5 Department of Anaesthesiology, University Medical Center Hamburg-Eppendorf, Martinistraße 52, 20246 Hamburg, Germany; 6 Anesthesia Department, Rotkreuzklinikum Munich, Nymphenburger Str. 163, 80634 Munich, Germany

**Keywords:** Implementation, CFIR, Feasibility, Pharmaceutical management, Qualitative, Medication reviews, Aged, Multiprofessional collaboration

## Abstract

**Background:**

Number of drugs are increasing with older age and present a risk factor for various adverse health outcomes. A comprehensive medication therapy management (MTM) before admission for elective surgery may help reduce unnecessary and potentially inadequate medications (PIM) and thus improve patient health. Our goal was to evaluate the implementation of PHAROS, a perioperative MTM intervention study, from the perspective of health care providers. The PHAROS intervention aimed to improve medication appropriateness in older inpatients at the outpatient / inpatient interface.

**Methods:**

We performed a qualitative interview study within a pilot intervention study comparing a comprehensive MTM with standard care in older inpatients (≥ 65 years) in Germany. Semi-structured interviews with health care professionals were performed from March to July 2021. The Consolidated Framework for Implementation Research (CFIR) was used to guide development of interview guide, data coding, analysis, and reporting of findings.

**Results:**

Ten health care professionals involved in the implementation of PHAROS were interviewed. Based on CFIR-constructs, facilitators included need for and meaningfulness of the intervention as well as positive and supportive cooperation within the project team. Implementation of MTM at the interface of inpatient to outpatient care before elective surgery was hampered by personal and organizational barriers as well as barriers resulting from broader health care structures in Germany. In particular, lack of documentation standards, missing compatibility with clinical workflow, difficulties in stakeholder engagement, as well as communication barriers between outpatient and inpatient care interfaces hindered implementation of the intervention.

**Conclusions:**

Further studies should consider focusing on facilitators to pharmaceutical implementations such as transparent and clear communication structures between stakeholders, standardization of medication documentation, and intervention structures that are adapted to hospital workflows.

**Trial registration:**

https://drks.de Identifier: DRKS00014621, this study was part of the PHAROS study.

**Supplementary Information:**

The online version contains supplementary material available at 10.1186/s12877-024-05652-4.

## Background

The worldwide demographic change has led to a rapid increase in the number of patients above 65 years. With older age, the risk for systemic diseases and multimorbidity [1] increases constantly and often leads to more frequent use of multiple drugs (i.e. multimedication, also known as polypharmacy [2, 3]). Multimedication is associated with higher rates of hospitalization [4] and a higher risk of surgery [5], also increasing the risk of postoperative complications in the elderly [6, 7]. In addition, research has shown that a substantial proportion of older adults take unnecessary medications or potentially inadequate medications (PIM), which can exacerbate negative health outcomes [8–12]. Individual measures (e.g. improving patient health literacy [13, 14]) as well as structural interventions (e.g. medication reviews (MR) in inpatient [15, 16] or outpatient [17, 18] settings) to improve this situation have been identified [19]. However, this research has focused on either sector separately, and we know little about the impact of a comprehensive medication therapy management (MTM) involving both inpatient (e.g., treatment in a hospital) and outpatient (e.g., treatment at primary care physician´s (PCP) practice) care settings. MTM, also known as comprehensive medication management (CMM), builds on MR but is more extensive and is followed by continuous patient care from a multidisciplinary team of health professionals [20, 21]. In Germany, a unique situation exists, where patient care is strictly divided into an inpatient and an outpatient sector. Since both sectors are structured very differently in terms of personnel, financing, and organization (e.g., concerning patient health record), and patients frequently move between inpatient and outpatient settings, research combining both sectors is crucial to improve sustainable patient care. In 2021, the electronic patient record was introduced in the German healthcare system to centralize patient health data and help improve information gaps between inpatient and outpatient settings. However, at the time this study was conducted, most patients did not yet have such a record and lack of technical requirements often hindered its utilization. Therefore, communication between inpatient and outpatient sectors is often done on paper and in different medical records systems. Beyond that, forwarding of information between sectors depends highly on the patient´s own initiative and ability to collect and collate all their health information. As a result, patient information can be lost, which in turn may increase the risk of medication errors for patients [22]. Accordingly, in a recent pilot study (PHAROS), we introduced a comprehensive MTM intervention introducing preoperative optimization of patient medication for older adults at the interface of inpatient and outpatient sector to improve patient medication appropriateness before elective surgery [23]. However, integrating new MTM routines into patient care can be challenging, and understanding potential barriers and facilitators to implementation should help to identify factors required for sustainable implementation. Recent studies in this context have shown a range of barriers (e.g., lack of resources, resistance to change) as well as facilitators (e.g., digitalization of records, patient´s health literacy) associated with implementation success [24–26]. Accordingly, in this qualitative study, we evaluated the implementation of the PHAROS intervention from the perspective of health care professionals (i.e. health care professionals (HCPs) directly involved in implementing PHAROS). Specifically, we focused on understanding barriers and facilitators to implementing a comprehensive MTM at the interface of inpatient / outpatient sector aiming at improving medication appropriateness in older adults.

## Methods

### Study design and setting

This was a qualitative study within a pilot intervention trial of a MTM of elderly patients in perioperative settings (≥ 65 years undergoing elective surgery, see PHAROS study [23]). We performed semi-structured qualitative interviews with HCPs to evaluate implementation of PHAROS. The PHAROS trial, including this qualitative study, was conducted at the University Medical Center Hamburg-Eppendorf, a German academic medical center. The study is reported in accordance with the Consolidated Criteria for Reporting Qualitative Research (COREQ) [27]; the corresponding checklist for our study can be found in Supplementary File 1.

### PHAROS trial

The aim of the PHAROS trials was to determine the impact of MTM on the medication appropriateness of older patients before elective surgery. We expected timely MTM and optimization of patient medication before surgery to have a positive effect on medication appropriateness as well as health-related outcomes (e.g., quality of life, medication adherence, health literacy). In addition, PHAROS aimed to secure continuity of care between inpatient and outpatient sectors by improving cooperation between hospital pharmacists, hospital ward physicians (HWPs), and PCPs. The PHAROS trial, a pilot sequential intervention study, comprised two phases: First, a control group was recruited from standard care to obtain the status quo. Second, after recruitment of the control group was completed, we recruited patients for the intervention group, as we expected the intervention to impact standard care processes. For both study groups HCPs contacted patients three weeks before surgery, on the day of hospital discharge, and three months after hospital discharge. At all three times, we collected health-related patient data which were assessed using paper-and-pencil questionnaires. For patients in the intervention group we integrated MTM in addition to standard care, including medication reviews and reconciliation as well as standardization of medication documentation. Medication reviews and reconciliation in the intervention group were performed within a week after patient recruitment and on the day of hospital admission, respectively. Patient medication data was gathered with a medication history sheet from several sources (e.g., patients, HWP, medication records). We planned to recruit patients at least three weeks before elective surgery to allow sufficient time for medication review and changes due to medication optimization. In the event of potentially inadequate medication, one of two hospital pharmacists involved in the PHAROS trial contacted the patient’s PCP, making recommendations for optimizing the medication. Before elective surgery, the PCP then decided whether to implement them. Also, HWP were contacted to include medication recommendations in patient discharge letters. See the PHAROS study protocol for detailed descriptions of population, recruitment, as well as data collection and analysis [23].

### Theoretical framework

We build on the Consolidated Framework for Implementation Research (CFIR) for data collection, analysis, and interpretation [28]. The CFIR comprises 39 different constructs in five domains, i.e. intervention characteristics, outer setting, inner setting, characteristics of individuals, and implementation process. It aims to analyze barriers and facilitators of implementation efforts. We used the CFIR interview guide tool to generate central structure and content for the interviews focusing on our study goals and relevant constructs [29]. Subsequently, we discussed and adjusted the interview questions for our purpose creating a semi-structured version of our interview guide. This process was guided by CB. Questions were primarily clustered following the CFIR and mostly open-ended. The interview guide was piloted conducting a pilot interview with a clinical colleague of MS not involved in the PHAROS project. Afterwards, interview questions were discussed resulting in further adjustments to interview question formats and structure. Overall, we aimed to evaluate potential barriers and facilitators to implementing PHAROS following the five domains of the CFIR (Supplementary File 2). Table 1 shows the interview questions. In our interviews, we focused on the following CFIR-domains: characteristics of individuals (constructs: knowledge and beliefs about the intervention, self-efficacy), intervention characteristics (constructs: design quality and packaging, complexity), outer setting (construct: patient needs and resources), inner setting (constructs: readiness for implementation, implementation climate, networks and communications, structural characteristics), and implementation process (constructs: engaging, planning).

**Table 1:**
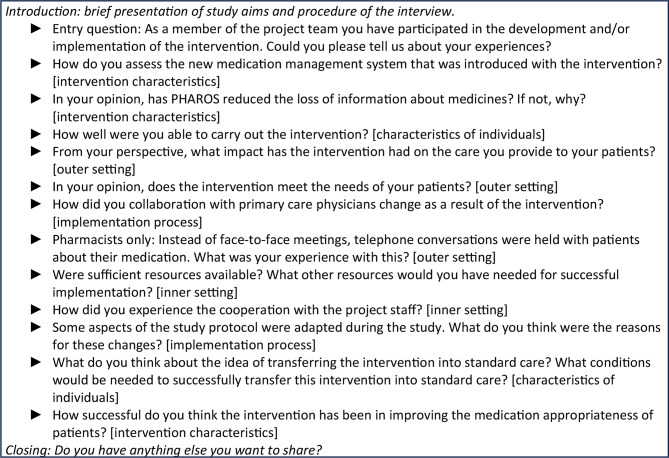
Interview questions

### Cohort and inclusion criteria

As per study protocol, we initially planned to interview HCPs as well as HWPs and surgeons treating patients in the intervention group to assess their experiences with the implementation of PHAROS. However, the PHAROS intervention could not be fully implemented during the study period and transition into standard care was not successful mainly due to communication difficulties with and between inpatient (i.e., HWPs) and outpatient (i.e., PCP) sectors. It also turned out that due to the difficult communication and a more than expected time required for the preoperative MTM, three weeks was not enough to make recommendations and optimize medication. Accordingly, even though many study patients were identified with potentially inappropriate medication, improvement of medication appropriateness could not be reached. Thus, staff not directly involved in the implementation of PHAROS were excluded since they often did not know about the PHAROS trial and were not able to give additional insight. Instead, qualitative interviews were conducted with HCPs only to examine barriers and facilitators during project planning and implementation.

### Data collection and analysis

Study participants were contacted by telephone or email and interviewed by telephone between March and July 2021 at their workplace. We invited all HCPs directly involved in the implementation of PHAROS to take part in an interview. We collected data until all potential interviewees had either participated or declined to participate in an interview. All interviews were conducted by a trained research assistant (MS), were audio-recorded, and transcribed verbatim. We followed simple transcription guidelines to increase readability [30]. In addition, MS took written field notes during the interview and filled out a postscript for each interview containing key aspects (e.g. interview length, prominent topics, interferences). For privacy reasons and due to small sample size, we did not collect any further demographic information and removed potentially identifiable information from transcripts before analysis. All transcripts were transferred to and analyzed with the coding software MAXQDA [31]. We analyzed the data using qualitative content analysis and used a deductive coding strategy to categorized identified barriers and facilitators according to CFIR domains [32]. MS coded all transcripts and discussed the coding process weekly with the principal investigator CB to ensure rigor. In case of discrepancies, data and emerging themes were discussed until consensus was reached. Relevant quotes were translated using DeepL. Due to the open-ended interview guide, interviewees were likely to refer to multiple constructs simultaneously, so that we allocated some statements to multiple CFIR-constructs where fitting.

### Reflexivity of research team

The primary research team of this qualitative study included CB and MS. The principal investigator (CB), a female professor for psychology and health care researcher with expertise in psychosocial outcome research. CB discussed findings with MS weekly and supervised the coding and interpretation process. A male PhD candidate and research assistant with a Diploma in Psychology (MS) created the interview guide as well as conducted and analyzed all interviews in consultation with CB. Previously, MS completed several training courses in qualitative research as part of his doctoral studies and gained experience in implementation and evaluation research as well as qualitative interviews and qualitative data analysis (MS). CB and MS were part of the PHAROS research team aiming at implementing the intervention. However, they were not involved in clinical patient care or implementation of the intervention but were responsible for evaluating the study. The remaining PHAROS research team was not involved in the coding process and data analysis of the findings but was responsible for development and implementation of PHAROS.

## Results

We contacted all HCPs (*n* = 11) directly involved in the implementation of PHAROS. Nobody rejected participation, but one person could not be reached and was therefore not interviewed. We detected that many aspects already mentioned by others were repeated after *n* = 8 interviews, indicating saturation of content. Most interviewees were female (90%) and worked in medical or pharmaceutical standard care in addition to project work (80%, Table 2). Two interviewees (20%) were medical students. Interviews lasted 18 to 51 min (median 32 min). Table 3 summarizes the facilitators and barriers identified by the project team during implementation of PHAROS. The domains of inner setting, implementation process, and outer setting were the most influential.

**Table 2:** Demographic characteristics of interviewees

Variable	*N* = 10
**Gender**	
Female	9 (90%)
Male	1 (10%)
**Profession**	
Pharmaceutical care	2 (20%)
Medical care	8 (80%)
**Professional experience**	
Junior (< 5 years post-qualification)	4 (40%)
Senior (≥ 5 years post-qualification)	4 (40%)
Medical student	2 (20%)

**Table 3:** Overview of barriers and facilitators

CFIR domain/construct	Facilitators	Barriers
**Characteristics of Individuals**		
Knowledge and beliefs about the intervention	• Health care professionals see sense and necessity of the intervention	• Transfer to standard care estimated to be difficult due to barriers in inner settings
Self-efficacy		• Performing the intervention more tedious and challenging than expected
**Intervention Characteristics**		
Design quality and packaging	• Instruments to analyze medication works fine	• Length and content of the patient questionnaire
Complexity		• Too many process steps and involved stakeholders to be efficient
**Outer Setting**		
Patient needs and resources	• General interest to participate in study	• General lack of eligible patients • Severity and consequences of diseases limits patient participation (e.g. limited mobility, limited cognitive function) • COVID-19 pandemic
Health and medication literacy	• Increased engagement with own health and medication through study participation	• Limited health literacy decreases interest and perceived benefit from participating in study
**Inner Setting**		
**Readiness for implementation**		
Available Resources	• Health care professionals´capacity for performing the intervention	• Limited time and availability of space to perform intervention
**Implementation climate** Compatibility		• Short term surgery planning limits medication adjustments before surgery • Frequent rotation of physicians between hospital wards • Lack of documentation of patient medication and too many available sources of documentation
**Networks and communications**	• Project management and coordination by the study nurse • Positive project teamwork and support	• Separation of working steps for clinic and pharmacy • Lack of communication and transparency about study responsibilities and process
**Structural characteristics**		• High number of stakeholders and intersections involved • Lack of connection between primary care and hospital
**Implementation Process**		
**Planning**		• Study protocol and intervention not planned in sufficient detail • Need to continuously adapt and expand the study protocol • Covid-19 pandemic
**Engaging**		
Primary care physicians	• Primary care physicians generally interested in the intervention	• Limited resources of primary care physicians hinder participation • Initiating contact to primary care physicians difficult • No feedback from primary care physicians on recommendations to change patient medication
Hospital ward physicians		• Missing hospital ward physicians´ involvement and interest in intervention • Hospital ward physicians did not give feedback to any of the recommendations on medication adjustments
Patients	• Acquiring patient medication history by telephone improved data collection	• Recruiting of patients very time-consuming and tedious

### Characteristics of individuals

There were several facilitators and barriers concerning the HCP´s individual characteristics. HCPs described the idea of the intervention (i.e., to improve medication appropriateness of elderly patients) as valuable and necessary, illustrating its need in the context of a growing older population (construct: knowledge and beliefs about the intervention).

However, many HCPs indicated that performing the intervention was more challenging than expected because the complexity of the process made it more difficult to implement (construct: self-efficacy; see also domain: intervention characteristics).

*“It still takes an incredible amount of effort on the part of the individual stakeholders to bring such a study to a successful conclusion.”* (S4P01).

### Intervention characteristics

The intervention included several measures to collect data on demographic patient characteristics, clinical aspects, pharmaceutical issues, and psychosocial outcomes.

On the one hand, project pharmacists reported that the instruments to collect and analyze patient medication were easy to use (construct: design quality and packaging).

On the other hand, patient questionnaires to collect data on psychosocial outcomes were criticized repeatedly for their length, structure, and content making it difficult for patients to answer (construct: design quality and packaging; see also domain: outer setting).

*“There were always so many follow-up questions that always referred to a sentence at the very beginning. And at some point*,* the patients no longer understood that this question still referred to something from a few pages earlier. […] But they were a lot like that*,* that you always had to explain how it was meant.”* (S4P08).

In addition, HCPs perceived the intervention as too complex and small-scale due to the potential involvement of many different stakeholders and their required cooperation (construct: complexity).

*“I can imagine it’s very complicated because you have so many different departments. The pharmacist*,* the primary care physician*,* then maybe the inpatient physicians here at the hospital*,* who then prepare the discharge medication. So*,* that’s something I can imagine. Once so many people are involved*,* it can just get very complicated.”* (S4P09).

### Outer setting

Patient needs and resources comprised several factors that HCPs perceived as facilitator or barrier. HCPs noted that patient interest in participating in the study to have their medication reevaluated was generally high (construct: patient needs and resources).

*“There is a lot of interest. Many patients actively ask for it or want their medication to be simplified and perhaps something to be deleted. And they are pleased that someone is really taking another structured look at it.”* (S4P04).

Another major topic was related to the patients´ health and medication literacy (construct: patient needs and resources). HCPs pointed out that the intervention could benefit patients by engaging more with their own health. For example, consultations with their physician or the hospital pharmacist during the intervention could help asking questions or clarifying uncertainties.

*“Yes*,* I think the patients were simply made aware that the topic is relevant. So that they perhaps also critically questioned themselves*,* how many medications am I actually taking and what is that doing to me? And what also became very clear through this questionnaire before the surgery was that they reflected on themselves: How well did the doctor explain the medication to me? Did he actually talk to me about side effects or about alternatives?”* (S4P10).

However, HCPs also perceived a potential imbalance concerning the patients´ health literacy and study participation. Project members felt that patients with limited health and medication literacy could benefit substantially from the intervention but were more likely not to participate because of these limitations, suggesting that patients may not be aware of the necessity to adjust their medication.

*“And I mean*,* we are dealing with health literacy in the project*,* and health literacy is a very important factor*,* especially with this medication. And that’s one of the main problems patients have*,* that they don’t know at all what [kind of medication] they’re getting. And what they’re taking and why.”* (S4P06).

Furthermore, there was one main factor regarding patient needs and resources that constituted a substantial barrier of implementation. It became apparent that the inclusion criteria of the study were primarily aimed at very ill patients, who, however, often would find it too strenuous to participate in the trial due to the severity of their illness. This was especially visible for the follow-ups. Accordingly, staff emphasized the substantial lack of eligible patients during their continuous recruitment efforts in the study.

*“Of course*,* we often got much sicker patients just to see an effect of the measures. We don’t have that many of them. The patients who are in such a bad shape that they could be included in the study often don’t feel like it because it’s exhausting for them.”* (S4P03).

### Inner setting

Available resources were a common concern among project members. While number of personnel was regarded sufficient to implement the intervention, staff expressed frustration concerning the limited time and space to conduct patient interviews and complete questionnaires (construct: available resources).

*“And as far as rooms are concerned. Rooms are a disaster here. We have far too few rooms and always have to fight for them.”* (S4P06).

Another major factor that complicated the implementation was the missing fit of the innovation with the hospital structures and work processes (construct: compatibility). First, planning of surgeries was often short-term due to changing priorities resulting in limited time to perform the intervention prior to surgery.

*“I think that’s also the case because it doesn’t correspond to the reality of how the process usually works. Because patients don’t usually come 21 days before surgery*,* and surgeries are scheduled on shorter notice.”* (S4P05).

Second, it was planned to involve HWPs in the study process so that they could include the medication recommendations in the discharge letter. However, this involvement could not be realized due to frequent staff rotation between wards and acted as a barrier (construct: compatibility; see also domain: implementation process).

*“When that conversation with the physicians comes up. This is simply complicated by the fact that we have such a high fluctuation of staff on the wards that the physicians sometimes rotate wards on a weekly basis.”* (S4P06).

Third, HCPs outlined potential barriers caused by lack of documentation of patient data potentially leading to errors in administration of medication (e.g., by simply adopting outdated medication from patient records; construct: compatibility).

*“We keep noticing inconsistencies. Maybe it’s just that the medications are adopted from previous stays*,* and that sometimes doesn’t match what the patients have told us they’re taking. Sometimes there are no medications entered at all. If patients are only there for one or two days*,* sometimes we see that nothing is entered at all.”* (S4P04).

In addition, lack of documentation standards and lack of digitalization constituted barriers and complicated reliable recording of patient medication.

*“Because I think nothing is worse than this sending of 20 pieces of paper back and forth. I write it on a piece of paper*,* send it to the pharmacy*,* they write me a piece of paper*,* I put it in the letter*,* they send it to the family doctor*,* the family doctor writes a new piece of paper for the patient.”* (S4P03).

HCPs highlighted the positive teamwork and support of the project team. However, lack of communication about work steps and responsibilities between different departments acted as a barrier throughout the project.

*“It was just that [department A] did one part independently and [department B] did another part independently. And because of that*,* we actually lost the connection points a little bit in some places. And it’s now split in two directions.”* (S4P06).

In addition, HCPs identified barriers resulting from existing patient care structures and different stakeholders involved in the project (construct: structural characteristics).

*“And in addition*,* we do not have a very good connection from the outpatient area to the inpatient area.”* (S4P03).

### Implementation process

Regarding the implementation process, two main constructs (i.e. planning and engaging) were very prevalent. HCPs stressed the need to continuously adapt and expand the study protocol due to unforeseen barriers in order to keep up implementation of the intervention. This was often attributed to inadequate planning of the study in advance.

*“If we had described more clearly what exactly we were doing*,* if we had specified more. We simply had very*,* very many discussions about what exactly that might look like - for example*,* we should have defined beforehand what exactly that should look like in practice. That would certainly have been helpful.”* (S4P06).

Inviting different individuals (i.e., patients, primary care physicians, hospital ward physicians) to participate in the intervention proved to be difficult. Primary care physicians, while showing a general interest in improving medication appropriateness of their patients according to the HCPs, could often not be reached and did not give feedback to any of the recommendations on medication adjustments within the project. HCPs suggested several reasons for this, including missing resources, uncertainty in prescribing other than routine medications, and a feeling of paternalism.

*“And that*,* I would imagine*,* is also one of the reasons that could possibly prevent the change in medication. Because then they have to get out of their familiar routine. And that’s understandable because the primary care physician has a routine with the medications he prescribes. He knows the side effects*,* he has been prescribing them for 10 years*,* he has had good experience with them. And now he is suddenly supposed to prescribe a drug that he doesn’t even know?”* (S4P01).

Contacting hospital ward physicians proved to be laborious and time-consuming. This was often explained by lack of interest or prioritization of the study among HWPs (construct: engaging; see also construct: compatibility):

*“But then getting my medical colleagues on the ward to do something with the information was impressively difficult.”* (S4P07) and *“We went to the departments and spoke to them ourselves. But there is no cooperation.”* (S4P02).

Similarly, engaging patients for the study turned out to be difficult and cumbersome including various methods to recruit patients and collect data (e.g. interviews during hospital stay, recruitment by telephone, data collecting through home visits).

*“I think the problem is the home visits*,* which are incredibly time-consuming. They were important for the study*,* but not feasible at all in reality.”* (S4P03).

### Covid-19 pandemic

Since the implementation of the intervention fell in the period of the Covid-19 pandemic, staff members also described their experiences during the two lockdowns in Germany. The pandemic rapidly changed hospital processes including scheduling of surgery and patient admissions.

Some project members described the uncertainty of planned surgeries in the beginning of the pandemic (construct: planning):

*“And then*,* of course*,* the stability of the surgery schedule was completely blown apart. And then it was always unclear which surgeries are important now? Which surgeries are taking place? What is an important surgery? What is a surgery that is not so important?”* (S4P01).

Additionally, a hospital wide restriction of patient contact for research purposes decreased the number of elective patients substantially.

*“Well*,* of course Corona has changed things. Last year*,* we actually had an admission stop as a result of Corona. We were not allowed to admit any more patients for clinical studies.”* (S4P04).

HCPs also realized that patients avoided hospital stays even after the lockdown was lifted because they still feared Covid-19 infection. This was seen as a continuous recruitment barrier (construct: patient needs and resources).

*“Even then*,* when we were able to start recruiting again*,* surgeries were postponed*,* patients were anxious*,* and didn’t want to come back.”* (S4P06).

## Discussion

From the perspective of HCPs, this qualitative study explored facilitators and barriers to implementing PHAROS, a pharmaceutical intervention which aimed at improving older patients´ medication appropriateness at the interface of outpatient to inpatient sector with focus on optimizing medication before elective surgery. Overall, the study shows that while the idea and need for such an intervention is apparent for most stakeholders, personal and organizational barriers to implementation constrained execution and thus transfer to standard care.

As involvement of different stakeholders at several cross-sectoral interfaces of patient care remains challenging, facilitators to implementation of a pharmaceutical intervention such as PHAROS include introducing easy and efficient communication structures as well as intervention structures consistent with different sectors of patient care (e.g., hospital, primary care). In addition, we found that missing standards of medication documentation limit structured data collection and management, constituting another implementation barrier.

Our results add to the research field of evaluating the implementation of interventions to improve medication appropriateness in older adults. One recent scoping review found 29 studies between 2010 and 2022 that conducted process evaluation of interventions to reduce PIM in older adults focusing on the implementation outcomes by Proctor et al. [24, 33]. The study found that multidisciplinary interventions were the most common. However, most of these interventions focused on primary or outpatient care and only few of them involved qualitative interviews to evaluate the implementation process. Nevertheless, the review identified several barriers and facilitators comparable to our study. For example, the idea and need to reduce PIM was generally perceived as important and meaningful, even though time constraints, workload, and communication between stakeholders often acted as barriers to implementing an intervention.

Medication documentation errors due to insufficient standardization were revealed as one major obstacle in the PHAROS trial. This is especially apparent when involving different medical specialists per patient or frequent patient transition between inpatient and outpatient sectors. These errors constitute a long-known quality management issue in patient care [34, 35]. Since 2016, for patients in Germany taking at least three systemic drugs, PCPs on request are required to issue a standardized medication plan [36]. However, our interviews suggest that medication plans are often incomplete still or even missing. Furthermore, our study shows that involvement of several health providers in patient care complicates coordination of different department-specific medications, and communication barriers appear to be highly prevalent in this area. This is in line with previous research [34, 37, 38]. For the successful implementation of a pharmaceutical intervention like PHAROS, a more direct and straightforward collaboration with individual primary care physicians and selected departments in the hospital could improve communication between stakeholders. In addition, establishing the use of automated electronic medication plans that can be viewed by all involved stakeholders may also be helpful.

Our interviews also addressed the question of whether patients with adequate health literacy would be more likely to participate in the study than patients with limited health literacy, even though patients with limited health literacy may benefit more from the intervention. Associations between health literacy and medication adherence have been confirmed in previous studies and improving health literacy could further help decrease medication errors in older patients [14, 39]. Finally, our interviews suggest that, although we aimed to include elderly polymedicated, multimorbid patients, barriers in the intervention design as well as implementation process may have prevented the most vulnerable of them from being included in PHAROS. Future studies should concentrate on how to successfully reach the most vulnerable of patients by involving them in the development of intervention study early on.

In summary, lack of standardization of medication documentation and missing collaboration between stakeholders were among the most prevalent barriers to implementing PHAROS. Reducing these barriers through careful planning and providing appropriate infrastructure appear to be essential for successful implementation of a pharmaceutical intervention like PHAROS. However, alternative patient care systems, e.g. by centralizing information that allows inpatient as well as outpatient care professionals to access patient information equally, could also be necessary to reduce complexity of communication between providers. The electronic patient record introduced in 2021, which will be mandatory for all insured persons in Germany from 2025, could help to close this gap.

### Strength and limitations

We included a sample of ten HCPs in our interview. Unfortunately, we were not able to collect experiences from HWPs and surgeons. As the intervention was not successfully implemented, neither HWPs nor surgeons were directly involved in implementing of PHAROS. This may have led to potential bias, since we only included the perspectives of staff members performing the implementation work. Also, the study sample was relatively small. However, we were able to interview all but one HCPs directly involved in implementing PHAROS. Since our aim was to explore possible facilitators and barriers to implementing PHAROS and to assess implementation success, experience from HCPs was the most crucial source of information.

Interviewed staff and interviewer (MS) were part of the same research team aiming at implementing PHAROS, which represents another limitation. Accordingly, staff might have been restrained or rated PHAROS more positively due to personal involvement. However, staff openly expressed negative experiences during the implementation, indicating a rather trusting atmosphere in the interviews. Also, most of the interviewed staff worked as clinical staff in standard care and were able to give additional insights into work processes and structures. In addition, MS worked strictly as a researcher and was not involved in clinical care of the patients allowing for a certain degree of neutrality. Nevertheless, coder bias still cannot be completely excluded. Also, due to the second Covid-19 lockdown in Germany, interviews were conducted by phone, which may constitute another limitation.

In addition, we did not interview patients included in the trial about their experience with PHAROS. Thus, we are not able to give insight into the patients´ perspectives. However, the project was implemented primarily through HCPs, and we are confident that we have identified the main barriers and facilitators through the interviews with them.

Strengths of this study were reliance on a solid theoretical basis and a priori defined constructs of interest following the CFIR. In addition, we were able to interview HCPs from different departments directly involved in the development and implementation of the project. Finally, to our knowledge, this is one of the first studies in this field to focus on the inpatient sector as well as outpatient sector, adding to findings of studies focusing on only one sector.

## Conclusions

In conclusion, necessary facilitators to successfully implement a pharmaceutical intervention to improve medication appropriateness in older inpatients before elective surgery seem to be transparent and regular communication between inpatient and outpatient sectors, as well as standardization of medication documentation, and simple intervention structures consistent with different workflows. Also, using a realistic intervention design, careful study planning, and sufficient resources to conduct studies are crucial facilitators.

## Electronic supplementary material

Below is the link to the electronic supplementary material.

Supplementary Material 1

Supplementary Material 2

## Data Availability

All data relevant to the study are included in the article or uploaded as supplemental information.

## Abbreviations

CFIR: Consolidated Framework for Implementation Research
CMM: Comprehensive medication management
HWP: Hospital ward physician
MR: Medication review
MTM: Medication therapy management
PCP: Primary care physician
PHAROS: Pharmaceutical management of elderly high-risk patients in perioperative settings
PIM: Potentially inadequate medications
