# Supplementary material for: Barriers and facilitators to the implementation of PHAROS, a perioperative pharmaceutical management intervention for older adults – a qualitative interview study from the perspective of healthcare providers
Source: BMC Geriatr. 2025 Jan 21;25:47. doi: 10.1186/s12877-024-05652-4 (PMC11748341; doi:10.1186/s12877-024-05652-4)
Supplement: Supplementary file 2 — Supplementary Material 2 [file 12877_2024_5652_MOESM2_ESM.docx]

**Supplementary File 2.** Domains and constructs from CFIR^1^ used for deductive analysis and interpretation

| **CFIR domain and constructs** | **Short Description** |
| --- | --- |
| **Characteristics of Individuals** |  |
| Knowledge and beliefs about the intervention | Individuals’ attitudes toward and value placed on the intervention as well as familiarity with facts, truths, and principles related to the intervention. |
| Self-efficacy | Individual belief in their own capabilities to execute courses of action to achieve implementation goals. |
| **Intervention Characteristics** |  |
| Design quality and packaging | Perceived excellence in how the intervention is bundled, presented, and assembled. |
| Complexity | Perceived difficulty of the intervention, reflected by duration, scope, radicalness, disruptiveness, centrality, and intricacy and number of steps required to implement. |
| **Outer Setting** |  |
| Patient needs and resources | The extent to which patient needs, as well as barriers and facilitators to meet those needs, are accurately known and prioritized by the organization. |
| **Inner Setting** |  |
| Readiness for implementation | Tangible and immediate indicators of organizational commitment to its decision to implement an intervention. |
| Implementation Climate | The absorptive capacity for change, shared receptivity of involved individuals to an intervention, and the extent to which use of that intervention will be rewarded, supported, and expected within their organization. |
| Networks and communications | The nature and quality of webs of social networks and the nature and quality of formal and informal communications within an organization. |
| Structural characteristics | The social architecture, age, maturity, and size of an organization. |
| **Implementation Process** |  |
| Engaging | Attracting and involving appropriate individuals in the implementation and use of the intervention through a combined strategy of social marketing, education, role modeling, training, and other similar activities. |
| Planning | The degree to which a scheme or method of behavior and tasks for implementing an intervention are developed in advance, and the quality of those schemes or methods. |

1. Damschroder LJ, Aron DC, Keith RE, et al. Fostering implementation of health services research findings into practice: a consolidated framework for advancing implementation science. *Implementation Science : IS* 2009;4:50. doi: 10.1186/1748-5908-4-50
